# Supplementary material for: Energy harvesting optical modulators with sub-attojoule per bit electrical energy consumption
Source: Nat Commun. 2021 Apr 19;12:2326. doi: 10.1038/s41467-021-22460-1 (PMC8055879; doi:10.1038/s41467-021-22460-1)
Supplement: Supplementary file 1 — Supplementary Information [file 41467_2021_22460_MOESM1_ESM.pdf]

Energy harvesting optical modulators with sub-attojoule per bit electrical energy consumption

Supplementary Information

M. de Cea,[1] A. H. Atabaki,[1] R. J. Ram[1]*

(1) Research Laboratory of Electronics, Massachusetts Institute of Technology,

Cambridge, MA 02139, USA.

* Email: rajeev@mit.edu

Supplementary Discussion 1. Photocurrent generation mechanisms in state-of-the-art Si modulators.

Our PV modulator concept applies to any optical modulator (regardless of material platform, structure, operational principle) that can act as a photovoltaic cell, i.e, that generates a photocurrent and develops a photovoltage when light is input to the device. The source of this photocurrent, either intentional or due to parasitic effects, is irrelevant for the operation of the device.

The presence of photocurrent in electroabsorption modulators is obvious, since these achieve modulation by the absorption of the input optical power when a '0' bit is transmitted, which generates electron-hole pairs in the device and a current as a result.

On the contrary, there is in principle no reason why electro-refraction modulators should generate a photocurrent since they rely on the change in refractive index with applied voltage and not on absorption mechanisms. In particular, Si modulators typically operate at wavelengths well below its bandgap (1300 nm and 1550 nm are the most common operating wavelengths), therefore linear optical absorption resulting in photogenerated current should be almost nonexistent.

There exist, nevertheless, several physical mechanisms in Si allowing for absorption of light with energy below the bandgap resulting in significant photocurrents [1]. In fact, several approaches exploiting these effects for power monitoring [2] [3], stabilization of microring resonators [4] and realization of photodetectors [5] have been reported in the literature.

Three different mechanisms contribute to photocurrent generation in Si optical waveguides:

1. Defect Mid-Bandgap Absorption (DMBA), which is due to the presence of crystalline defects or foreign atoms in the bulk of the Si waveguide, which break the crystal periodicity and generate states with energy levels within the bandgap. By intentionally adding defects into a silicon waveguide through ion implantation, mid-IR photodetectors with responsivities as high as 0.8 A/W over a wavelength range from 1270 nm to 1700 nm have been realized [6].
2. Surface State Absorption (SSA). The termination of the crystal structure at the surface of a semiconductor distorts its band structure and creates intra-gap states [7], which can then result in sub-bandgap light absorption. Such effect has been reported, characterized and exploited in standard silicon photonic waveguides [8] [9] [10].
3. Two photon Absorption (TPA), where the simultaneous absorption of two photons results in the generation of an electron-hole pair. Being a nonlinear effect, TPA becomes important at high input optical powers or in resonant structures.

At moderate input optical powers, SSA is believed to be the dominant photocurrent generation mechanism in modern silicon photonic waveguides over DMBA. This is because the high quality of the fabrication process results in a low density of defects in the bulk of the Si waveguide, and thus reduced DMBA. Two main factors dominate the strength of SSA in optical waveguides: (1) The overlap of the optical mode with the silicon surface, and (2) the quality of the surface passivation [11]. SSA does not show a strong wavelength dependence in the wavelength ranges of interest [12] [13].

Besides absorption mechanisms intrinsic to the silicon waveguide, another approach for photocurrent generation is the addition of other materials with a smaller bandgap such as germanium (Ge), which usually requires the use of modified silicon photonic processes since pure Ge is not a standard layer in CMOS foundries. Nonetheless, silicon-germanium (SiGe) is used in standard CMOS to strain the channel of pFET transistors, which can be used to increase optical absorption in unmodified CMOS platforms [14]. It is important to note that this is only possible for O band wavelengths and below, since the absorption edge of SiGe alloys is near 1300 nm [15].

We expect, then, photogenerated current to be present in most modern optical modulators in general, and in Si modulators in particular. In Supplementary Discussion 2 we show experimental measurements for the photogenerated current due to optical absorption in two different silicon resonant modulators.

Estimating photocurrent generation in state-of-the-art silicon modulators due to SSA

As mentioned above, several approaches exploiting SSA for optical power monitoring in integrated silicon devices have been reported in the literature. We can use these works to estimate the SSA-generated photocurrent in the high-performance Si modulators reported in the literature (Supplementary Table 1). We selected the publications employing the most similar fabrication techniques and device geometries as the modulators in Supplementary Table 1 and derived an approximate value for the photocurrent generating SSA absorption parameter α_{SSA}. These are shown in Supplementary Table 4, where we can see how values for α_{SSA} are reasonably consistent over published works. A conservative estimate seems to be $\alpha_{SSA} = 1\ m^{-1}$, which translates into a loss of 0.04 dB/cm.

Using this value for α_{SSA} we can get an estimate for the responsivity of the Si modulators in Table 1 from the reported device geometries and Q factors (in the case of resonant devices) [1]. The extracted responsivity values are shown in Supplementary Table 5, along with the photocurrent and associated DC energy dissipation for the input optical powers that were used to experimentally characterize the devices. Responsivities around 1 mA/W and photocurrents close to 1 µA are reached. As shown, the energy dissipation associated with the photocurrent is on the order of a few percent of the total electrical power consumption.

As shown in Supplementary Discussion 2, experimental measurements in fabricated devices are consistent with the derived value of R ≈ 1 mA/W.

[1] We can estimate the quantum efficiency as $\eta = 1 - exp(-\alpha_{SSA} L_{eff})$. For Mach-Zehnder modulators, L_{eff} corresponds to the device length L. In the case of resonant devices, $L_{eff} = F\ L/2\pi$, where F is the finnesse of the resonance.

Supplementary Discussion 2. Photocurrent generation in state-of-the-art Si modulators: Experimental results.

Supplementary Fig. 1 shows measured photovoltaic responses (IV curves, open circuit voltages and short circuit currents) as a function of input optical power in the waveguide for two of our CMOS resonant optical modulators. Blue curves correspond to a Si-only device (see [16] for the device design and characterization) where photocurrent generation is primarily due to SSA. Orange curves show the response for the silicon resonator incorporating a SiGe band, which is the device we used for our experimental demonstration of photovoltaic modulation.

We measure a responsivity R = 0.85 mA/W for the Si-only modulator, which is in good agreement with the 1 mA/W estimate we derived in Supplementary Discussion 1. As expected, the addition of SiGe results in an increased responsivity R = 34 mA/W due to absorption from phonon-assisted indirect electronic transitions at wavelengths around 1280 nm [15]. Open circuit voltages above 0.5 V and photogenerated currents on the order of 1 μA for small input optical powers on the order of 10 μW are measured.

For the silicon resonator incorporating a SiGe band, we measure a total waveguide loss of 27.5 dB/cm. From the total, 2.6 dB/cm (0.6 cm^{-1}) correspond to phonon-assisted absorption resulting in the generation of photocurrent. The additional 24.9 dB/cm loss are mainly from free carrier absorption due to the presence of doped regions in the areas where the optical mode is propagating.

Supplementary Discussion 3. PV modulator small signal model.

The small signal equivalent circuit of the PV modulator is shown in Supplementary Fig. 2(a), which corresponds to that of a common source amplifier with the modulator as the load [17]. As mentioned in the main text, a voltage gain given by $A_v = \Delta V_{\mathrm{mod}}/\Delta V_{\mathrm{gs}} = -g_m(r_0||r_d||r_{\mathrm{pc}})$ at DC frequencies is achieved. It is also clear from the circuit in Supplementary Fig. 2(a) that the device bandwidth is limited by the RC time constant at the modulator terminals, which is $\tau = (C_{\mathrm{mod}} + C_{\mathrm{ds}})(r_0||r_d||r_{pc}) \approx C_{\mathrm{mod}}(r_0||r_d)$. $g_m = dI_{\mathrm{sw}}/dV_{\mathrm{gs}}$ is the transistor transconductance, $r_0 = dV_{\mathrm{mod}}/dI_{\mathrm{sw}}$ the output resistance of the transistor and $r_d = dV_{\mathrm{mod}}/dI_{\mathrm{mod}}$ the dynamic resistance of the diode. $r_{\mathrm{pc}} = (dI_{\mathrm{pc}}/dV_{\mathrm{mod}})^{-1}$ accounts for the change in the generated photocurrent with the voltage at the modulator terminals. Notice how in the main text, we considered $r_{\mathrm{pc}} \gg r_0||r_d$ so that $(r_0||r_d||r_{\mathrm{pc}}) \approx (r_0||r_d)$. We will make the same assumption in this section. Supplementary Discussion 8 discusses the validity of such an approximation.

Supplementary Fig. 2(b) shows the achieved voltage gain A_v as a function of bias voltage, which shows that gains between 10 and 15 are achievable independent of photocurrent. Good agreement between experimental and simulation results is obtained, although we measured a lower small signal electrical gain than that predicted by simulation. Experimental data characterizing the fabricated transistors suggest that this is due to g_m being about 25% lower experimentally. We observed a stronger dependence of g_m on the transistor drain-source voltage (V_{mod} in our configuration) than that predicted by simulations, which explains why larger deviations are obtained for lower generated photocurrents.

The evolution of the gain A_v and the bandwidth at the bias point that results in the maximum voltage gain is shown in Supplementary Fig. 2(c), which reproduces Fig. 3(b) in the main text. To explain the observed dependence with I_{pc} it is of interest to study how the relevant parameters affecting these metrics, namely g_m, R = $(r_0||r_d)$ and C_{mod} depend on photocurrent. This is depicted in Supplementary Fig. 2(d).

Several observations can be made:

1. The transistor transconductance g_m increases with increasing photocurrent. This is because the current flowing through the transistor I_{sw} at the maximum gain point increases with larger

I_{pc}, and in the weak inversion regime $g_m \propto I_{\text{DS}} = I_{\text{sw}}$ [17]. Operation of the transistor in weak inversion is desirable because it results in maximum gain.

2. At the maximum gain point, the system resistance R = $(r_0||r_d) \approx r_0$. This makes the small signal gain depend solely on the transistor characteristics $|A_{v,\text{max}}| \approx g_m r_0$.
3. R $\approx r_0$ decreases with increasing photocurrent. This is because $r_0 \propto 1/I_{\text{sw}}$, and as we discussed above larger I_{pc} result in larger I_{sw} at the maximum gain point.
4. The modulator capacitance C_{mod} does not significantly vary with photocurrent.

Through these observations we can explain the evolution of A_v and bandwidth shown in Supplementary Fig. 2(c). The maximum gain $|A_{v,\text{max}}| \approx g_m r_0$ is mostly independent of photocurrent due to the fact that $g_m \propto I_{\text{sw}}$ and $r_0 \propto 1/I_{\text{sw}}$. The device bandwidth increases with photocurent due to the decrease in R and the fact that C_{mod} is approximately constant.

Independent of the value of I_{pc}, at the maximum gain point the device has a limited speed with a 3 dB bandwidth lower than 200 MHz, mainly caused by the large r_0 of the transistor, which is on the order of MΩ as shown in Supplementary Fig. 2(d). It is of interest to explore design possibilities to decrease the output resistance r_0 and improve the frequency response of the device. The output resistance of the transistor is given by:

$$r_0 = \frac{1}{\lambda I_{\text{DS}}} \tag{1}$$

Above, I_{DS} is the current flowing through the transistor (which corresponds to I_{sw} in our schematic), and λ is known as the channel length modulation parameter, which depends mostly on intrinsic technology parameters [2].This means that, unfortunately, there is no design approach that can reduce the value of r_0 for a given I_{sw}, which points to the conclusion that the bandwidth limitation is intrinsic to the PV modulator.

Nevertheless, and as discussed in the main text, larger bandwidths can be achieved in PV modulators at the cost of increased energy dissipation. This is because a strong gain-bandwidth tradeoff exists: a larger r_0 will yield a larger small signal gain ($A_v \approx g_m r_0$), but will result in a decreased bandwidth $\left(f_{3dB} \approx 1/(2\pi r_0 C_{\text{mod}})\right)$.

Supplementary Fig. 3 illustrates such trade-off (along with Fig. 3(c) in the main text). The gain and bandwidth as a function of gate bias for different photocurrents is shown in Supplementary Fig. 3(a). The gain-bandwidth product is shown in Supplementary Fig. 3(b) for different generated

photocurrents. Clearly, the PV modulator can be biased at a point with a smaller gain but a faster frequency response, which can be desirable in applications requiring high data rate communication.

To understand the gain-bandwidth tradeoff it is informative to study the evolution of the small signal parameters with bias voltage. This is shown in Supplementary Fig. 3(c) for a fixed photocurrent I_{pc} = 1 µA. Because $g_m \propto I_{\mathrm{DS}} = I_{\mathrm{sw}}$, an increase in g_m is observed as V_{gs} rises due to the fact that a larger current is flowing though the transistor. For large values of V_{gs}, channel length modulation (CLM) effects (the dependence of the transistor channel length on the drain-source voltage $V_{\mathrm{ds}} = V_{\mathrm{mod}}$ in our circuit) become important and g_m decreases as a consequence. Since $r_0 \propto 1/I_{\mathrm{sw}}$, a decrease is observed with increasing V_{gs} because of the rise in the current flowing through the transistor. The diode resistance $r_d \propto 1/I_{\mathrm{mod}}$ follows the opposite trend: an increase in r_d is observed with increasing V_{gs}, which is explained because the current flowing through the diode decreases as V_{gs} rises. As a consequence, the equivalent resistance at the modulator terminals $(r_0||r_d)$ is dominated by r_d at low values of V_{gs}, and by r_0 for large V_{gs}, as can be seen in the bottom plot of Supplementary Fig. 3(c).

These observations allows us to describe the evolution of the curves in Supplementary Fig. 3(a): For a fixed photocurrent, an increase in A_v is observed for growing V_{gs} due to an increase in g_m, until CLM effects decrease the gain. The decrease in R with bias voltage explains why we observe a larger bandwidth with increasing V_{gs}.

Several trends are observed in Supplementary Fig. 3(b): (1) the gain-bandwidth product increases as gate bias increases until it saturates at a certain value; and (2) the gain-bandwidth product is larger for larger generated photocurrents.

Both observations can be explained by noting that the gain-bandwidth product is simply given by:

$$|A_v| f_{3dB} = g_m (r_0||r_d) \frac{1}{2\pi (r_0||r_d) C_{\mathrm{mod}}} = \frac{g_m}{2\pi C_{\mathrm{mod}}} \qquad (2)$$

As shown in Supplementary Fig. 3(c), g_m increases as the gate bias voltage increases for a fixed photocurrent, which explains observation (1) above. Supplementary Fig. 3(d) shows how larger photocurrents result in larger g_m values, which explains observation (2).

[2] $\lambda \propto \sqrt{t_{ox} X_j}/L$, where t_{ox} is the gate oxide thickness, L is the gate length and X_j is the drain junction depth [17].

Supplementary Discussion 4. Equivalent gate capacitance in PV modulators.

To obtain the electrical energy that the external source needs to deliver to drive the PV modulator, an accurate calculation of the input capacitance of the device is essential. Clearly, this input capacitance corresponds, in a PV modulator, to the gate capacitance of the transistor.

From the PDK of the CMOS process we used to fabricate our experimental device, the physical gate capacitance is $C_{g,\mathrm{phys}} = C_{\mathrm{gb}} + C_{\mathrm{gs}} + C_{\mathrm{gd}} \approx 1.3$ fF. Nevertheless, the voltage gain obtained in the transistor due to its common source configuration results in an increase in the input capacitance seen by the driving electrical source, resulting in $C_g = C_{\mathrm{gb}} + C_{\mathrm{gs}} + (1 + |A_v|)C_{\mathrm{gd}}$, where A_v is the voltage gain achieved in our configuration. This increase in input capacitance is known as the Miller effect [17].

As a result of the Miller effect, the electrical energy dissipation in our modulator is given by $E_{\mathrm{el}} = \left[C_{\mathrm{gb}} + C_{\mathrm{gs}} + (1 + |A_v|)C_{\mathrm{gd}}\right]V_{\mathrm{pp}}^2/4$. As is apparent in Supplementary Fig. 2(b), it is important to recognize that the voltage gain A_v is strongly dependent on the voltage at the transistor gate V_{gs}. To accurately calculate the electrical energy consumed in our device we can define an effective gain $A_{v,\mathrm{eff}}$:

$$\mathrm{A_{v,eff}} = \frac{1}{V_{\mathrm{gs,max}} - V_{\mathrm{gs,min}}} \int_{V_{\mathrm{gs,min}}}^{V_{\mathrm{gs,max}}} A_v\left(V_{\mathrm{gs}}\right) dV_{\mathrm{gs}} \tag{3}$$

With this definition, the energy delivered by the external electrical source to drive the modulator is given by $E_{\mathrm{el}} = \left[C_{\mathrm{gb}} + C_{\mathrm{gs}} + \left(1 + \left|A_{v,\mathrm{eff}}\right|\right)C_{\mathrm{gd}}\right]V_{\mathrm{pp}}^2/4$. Notice we are assuming that the physical capacitances $C_{\mathrm{gb}}, C_{\mathrm{gs}}$ and C_{gd} are constant with voltage, which is a valid approximation when the device is operated near the maximum gain point in the subthreshold regime. From the PDK of the CMOS process, our transistor has $C_{\mathrm{gb}} = 0.3\,fF$, $C_{\mathrm{gs}} = 0.6\,fF$ and $C_{\mathrm{gd}} = 0.4\,fF$ at typical operating points of the PV modulator. These values do not depend strongly on the generated photocurrent, and are the values used to calculate the energy consumption reported throughout this work.

Notice how, in general, $A_{v,\mathrm{eff}}$ will be dependent both on the amplitude of the driving signal $V_{\mathrm{gs,pp}} = V_{\mathrm{gs,max}} - V_{\mathrm{gs,min}}$ and its DC bias $V_{\mathrm{gs,DC}} = \left(V_{\mathrm{gs,max}} + V_{\mathrm{gs,min}}\right)/2$. Supplementary Fig. 4 shows the equivalent input capacitance $C_g = C_{\mathrm{gs}} + \left(1 + \left|A_{v,\mathrm{eff}}\right|\right)C_{\mathrm{gd}}$ as a function of the amplitude of the driving signal $V_{\mathrm{gs,pp}}$ assuming that the device is biased at the voltage $V_{\mathrm{gs,DC}}$ that results in the

maximum voltage gain. Both the C_g inferred from the circuit simulations and that inferred from experimental measurements of the gain are shown. Since the experimentally measured gain is lower than that predicted by simulations (Supplementary Fig. 2(b)), so is the equivalent input capacitance C_g. We can see how the capacitance decreases as the peak to peak voltage increases, indicating a decrease in $A_{v,\text{eff}}$. This is because the voltage gain A_v decreases very rapidly when the gate voltage is not at the optimum value (Supplementary Fig. 2(b)).

Supplementary Discussion 5. Calculation of optical modulation performance in PV modulators.

To evaluate the optical performance of PV modulation we will consider a resonant modulator. A similar approach can be used to evaluate PV modulation in Mach-Zehnder structures.

As in a conventional silicon modulator, the voltage swing at the modulator V_{mod} changes the electron and hole density in the silicon and hence the refractive index of the optical resonator via the plasma dispersion effect [18], resulting in a shift in the resonance wavelength of the device, $\Delta\lambda_0$:

$$d\lambda_0 = \frac{d\lambda_0}{dV_{\mathrm{mod}}}\frac{dV_{\mathrm{mod}}}{dV_{\mathrm{gs}}}dV_{\mathrm{gs}} = \left.\frac{d\lambda_0}{dV_{\mathrm{mod}}}\right|_{V_{\mathrm{mod,bias}}(V_{\mathrm{gs,bias}})} A_v\left(V_{\mathrm{gs,bias}}\right)dV_{\mathrm{gs}} \quad (4)$$

Above, $d\lambda_0/dV_{\mathrm{mod}}$ is the modulation efficiency of the optical modulator (which we call intrinsic modulation efficiency), and A_v is the electrical gain of the PV modulator. We have explicitly indicated that both the intrinsic modulation efficiency and the gain depend on the bias point.

Supplementary Fig. 5(a) shows the results of evaluating Supplementary Eq. (4) using A_v given by the circuit simulation (Supplementary Fig. 2(b)) and representative values for $d\lambda_0/dV_{\mathrm{mod}}$ based on experimental results (shown in the inset of Supplementary Fig. 5(a)). Even for small photocurrents, shifts larger than 200 pm/V are achievable, which is >10x larger than the 20 pm/V achievable with conventional modulation (dashed line in Supplementary Fig. 5(a)). Such enhancement is due to the electrical gain achieved in PV modulation.

It is not obvious why larger photocurrents should result in larger modulation efficiencies, since we argued how the maximum electrical gain A_v is independent of I_{pc} (Supplementary Fig. 2(c)). Nevertheless, larger intrinsic modulation efficiencies $d\lambda_0/dV_{\mathrm{mod}}$ are achieved with larger photocurrents (Supplementary Fig. 5(b)). This is because the operating voltage at the modulator terminals V_{mod} increases, which sets the operating point of the diode in the weak forward bias, where modulation efficiency is larger than in reverse bias (inset of Supplementary Fig. 5(a)).

Note how the point of maximum modulation efficiency does not coincide with the bias that gives a larger electrical gain (dashed lines in Supplementary Figs. 5(a, b)). This is because larger intrinsic modulation efficiencies can be achieved at bias voltages that do not yield optimal electrical gain.

The total wavelength shift for a voltage swing at the input of the PV modulator between $V_{\mathrm{gs,min}}$ and $V_{\mathrm{gs,max}}$ is obtained by integrating Supplementary Eq. (4):

$$\Delta\lambda_0 = \int_{V_{\mathrm{gs,min}}}^{V_{\mathrm{gs,max}}} \left.\frac{d\lambda_0}{dV_{\mathrm{mod}}}\right|_{V_{\mathrm{mod}}(V_{\mathrm{gs}})} A_v(V_{\mathrm{gs}})dV_{\mathrm{gs}} \tag{5}$$

Note how, in general, $\Delta\lambda_0$ will depend not only on the amplitude of the applied voltage $V_{\mathrm{gs,pp}} = V_{\mathrm{gs,max}} - V_{\mathrm{gs,min}}$, but also on the DC voltage $V_{\mathrm{gs,DC}} = (V_{\mathrm{gs,max}} + V_{\mathrm{gs,min}})/2$. Supplementary Fig. 5(c) shows the maximum achievable $\Delta\lambda_0$ as a function of $V_{\mathrm{gs,pp}}$, assuming that the optimal $V_{\mathrm{gs,DC}}$ is chosen. For driving voltages < 100 $\mathrm{mV_{pp}}$, 10-40x larger resonance shifts are achieved when compared to conventional modulation (dashed line in Supplementary Fig. 5(c)). As expected, larger photocurrents result in larger resonance shifts for the same $V_{\mathrm{gs,pp}}$ because of the larger modulation efficiencies achieved.

A saturation of $\Delta\lambda_0$ is observed for larger driving voltages. This is because the maximum voltage swing at the modulator terminals corresponds to the difference between the open circuit and short circuit conditions $\Delta V_{\mathrm{mod,max}} = V_{\mathrm{oc}} - V_{\mathrm{sc}} = V_{\mathrm{oc}} - 0 = V_{\mathrm{oc}}$. Therefore, the maximum achievable resonance shift in the PV modulator is given by:

$$\Delta\lambda_{0,\mathrm{sat}} = \int_{0}^{V_{\mathrm{oc}}} \left.\frac{d\lambda_0}{dV_{\mathrm{mod}}}\right|_{V_{\mathrm{mod}}} dV_{\mathrm{mod}} \tag{6}$$

This is shown in Supplementary Fig. 5(d) as a function of photocurrent. An increase in $\Delta\lambda_{0,\mathrm{sat}}$ is observed with increasing photocurrent due to an increase in the open circuit voltage. It is important to note that conventional modulation does not have such limitation in the maximum achievable wavelength shift. Thus, for large peak to peak driving voltages (> 500 $\mathrm{mV_{pp}}$) conventional modulation will result in larger resonance shifts compared to PV modulation, and therefore better modulation performance. In other words, the use of PV modulation is the most advantageous for low peak to peak driving voltages < 100 $\mathrm{mV_{pp}}$, where there is no saturation of $\Delta\lambda_0$.

Modulation performance

From the resonance wavelength shift data in Supplementary Fig. 5(c) we can easily extract the modulation characteristics if we know the resonance shape T(λ) of our modulator.

Here, we assume a Lorentzian resonance, which is described in terms of its quality factors Q. The total Q factor of the resonance, usually called loaded Q (Q_{loaded}), is the inverse sum of the Q factors associated with each loss mechanism in the ring: $Q_{\mathrm{loaded}}^{-1} = Q_{\mathrm{coup}}^{-1} + Q_{\mathrm{int}}^{-1}$. Q_{coup} is associated with the optical loss due to the coupling of light from the ring to the output bus waveguide, and Q_{int} with the internal loss mechanisms in the ring, which are mainly radiation, scattering and absorption. The optical transmission through the ring is then given by:

$$T(\lambda_l) = \frac{\left(1-\frac{\lambda_0}{\lambda_l}\right)^2 + \frac{1}{4Q_{\mathrm{coup}}^2} + \frac{1}{4Q_{\mathrm{int}}^2} - \frac{1}{2Q_{\mathrm{coup}}Q_{\mathrm{int}}}}{\left(1-\frac{\lambda_0}{\lambda_l}\right)^2 + \frac{1}{4Q_{\mathrm{coup}}^2} + \frac{1}{4Q_{\mathrm{int}}^2} + \frac{1}{2Q_{\mathrm{coup}}Q_{\mathrm{int}}}} \tag{7}$$

Above, λ_0 is the resonance wavelength of the ring and λ_l is the laser wavelength. When Q_{coup} = Q_{int} the ring is said to be in the critical coupling condition, and the transmission is T=0 on resonance (λ_l=λ_0).

With T(λ) and the resonance wavelength shift for a given input signal at the transistor gate we can easily extract the modulation characteristics, namely Extinction Ratio (ER) and Insertion Loss (IL):

$$ER = |T(\lambda_l) - T(\lambda_l - \Delta\lambda_0)| \tag{8}$$

$$IL = max[T(\lambda_l), T(\lambda_l - \Delta\lambda_0)] \tag{9}$$

Note how different ER and IL can be achieved for a given $\Delta\lambda_0$ depending on the operating laser wavelength λ_l. For the figures shown in the main text (Fig. 2(d-g)) and the figures below, we choose the operating wavelength λ_l that results in the maximum ER, subject to the limitation that IL < 6 dB.

Besides the Q factors and the operating wavelength λ_l, the modulation performance is dependent on the photocurrent I_{pc}, the DC bias voltage $V_{\mathrm{gs,DC}}$ and the peak to peak voltage $V_{\mathrm{gs,pp}}$, whose combination determines the achieved wavelength shift $\Delta\lambda_0$ as described in above. Additionally, both $V_{\mathrm{gs,DC}}$ and I_{pc} determine the 3 dB bandwidth of the device.

Thus, given a resonance shape T(λ) and a generated photocurrent I_{pc}, varying $V_{\mathrm{gs,pp}}$ and $V_{\mathrm{gs,DC}}$ will change $\Delta\lambda_0$ and as a consequence the modulation ER. We can thus obtain surfaces of the modulation ER in terms of energy dissipation (which is directly translated to $V_{\mathrm{gs,pp}}$ through $E = C_g V_{\mathrm{pp}}^2/4$) and bandwidth (which is set by $V_{\mathrm{gs,DC}}$).

Two of such surfaces are shown in Supplementary Figs. 5(e, f) for a photocurrent I_{pc}= 1 μA. Supplementary Fig. 5(e) corresponds to a resonance with Q_{int} = 11,000, Q_{coup} = 13,000, Q_{loaded}= 6,000, while Supplementary Fig. 5(f) corresponds to Q_{int} = 22,000, Q_{coup}= 26,000, Q_{loaded} = 12,000. These are the same Q factors used for Fig. 2(d-g) in the main text.

As expected, the largest ER are achieved for the highest energy dissipation (i.e, a larger peak to peak driving voltage) and the lowest bandwidth (where larger electrical gains are obtained). Increasing bandwidths result in smaller ER because the small signal gain A_v decreases, and therefore a smaller $\Delta\lambda_0$ is obtained for the same driving signal. Similarly, larger Q factors achieve better performance because T(λ) is narrower, and therefore the same $\Delta\lambda_0$ results in a larger change in transmission. Increasing the photocurrent I_{pc} results in an overall upwards shift of the ER surface due to the larger $\Delta\lambda_0$ achievable.

The plots shown in Fig. 3(d-g) in the main text correspond to slices of the surfaces in Supplementary Figs. 5(e, f) along the energy axis.

Supplementary Discussion 6. Bit error rate of low extinction ratio modulation at sub-GHz speeds.

In conventional, high-speed optical communications, modulation with ER > 2 – 3 dB is usually required to establish close to error-free communication. Nevertheless, operation at the low bandwidths (Δf < 100 MHz) that our target applications require significantly change the performance tradeoffs. In particular, the low bandwidth requirements substantially decrease the equivalent noise power of the receiver chain detecting the modulated signal generated by our PV modulator. This allows for the achievement of close to error-free transmission with the ER levels (< 1 dB) we demonstrate in our work. In telecommunications applications, error-free communication is generally assumed for BER < 10^{-9}, while data communication applications usually require a BER < 10^{-12}.

To obtain the bit error rate (BER) characteristics of our optical link, we need to obtain the signal-to-noise ratio (SNR) at the receiver. Such SNR will depend on the particular receiver architecture, but we can estimate achievable BER based on reference system designs and scenarios. As we derive below, operating at bandwidths Δf < 100 MHz enables close to error-free communication with ER on the order of 0.5 dB for received optical powers above ~ 2 μW. For reference, the received optical power in our experimental demonstration was 3.75 μW (subtracting optical losses associated to the coupling from the chip to the output fiber).

Scenario 1: SNR limited by receiver electrical noise

This is the most common scenario in practical receiver implementations and corresponds to the situation where the total noise at the receiver site is dominated by the electrical noise of the analog electrical chain (used for transduction, amplification and processing of the received signal) present after photodetection.

To evaluate the BER in this scenario, we can obtain representative values for the equivalent noise at the input of the receiver by looking at commercially available options with bandwidths in the 100 MHz range.

In this scenario, the BER is given by:

$$BER = \frac{1}{2}\text{erfc}\left(\frac{P_1 - P_0}{2\sqrt{2}\sigma}\right) \tag{10}$$

Where P_1 (P_0) is the received optical power of the '1' ('0') bit, σ is the total noise equivalent power integrated over the receiver bandwidth and erfc is the complementary error function. By recognizing that $P_0 = P_1/ER$, we can rewrite Supplementary Eq. (10) as:

$$BER = \frac{1}{2}\text{erfc}\left(\frac{P_1(1 - 1/ER)}{2\sqrt{2}\sigma}\right) \tag{11}$$

Supplementary Fig. 6(a) shows the BER as a function of ER for different received optical powers for the '1' bit (P_1) for a receiver with a noise equivalent power (NEP) of 2.5 pW/$\sqrt{\text{Hz}}$ integrated over a bandwidth Δf = 100 MHz ($\sigma = NEP * \sqrt{\Delta f}$ = 25 nW). Such NEP is representative of a variety of optical receivers and transimpedance amplifiers with bandwidths in the 100 – 250 MHz range [19] [20] [21] [22]. Clearly, for received powers above 2 μW, error-free communication can be established with ER < 1 dB.

Scenario 2: Shot-noise limited system

Another possible scenario is to consider a shot-noise limited system, i.e, a system where the dominant source of noise is that associated with the power of the optical signal itself. While this is not the typical situation in practical optical links, shot-noise limited receivers are achievable for large received optical powers (usually > 1 mW).

In this case, the current noise variance at the detector is given by $\sigma^2 = 2qI\Delta f$, where q is the electron charge, Δf is the photodetector bandwidth and I is the photocurrent generated by the input optical signal, $I = RP_{\text{rec}}$. In the latter, R is the responsivity of the photodetector.

If we follow a similar approach as in scenario 1 above, but now account for the fact that the noise power is itself dependent on the power of the received signal (which is different for the '1' and '0' bit), we can obtain an expression for the achievable BER as a function of ER[3]:

$$BER = \frac{1}{2}\text{erfc}\left(\sqrt{\frac{RP_1}{q\Delta f}}\frac{(1 - 1/ER)}{2}\right) + \frac{1}{2}\text{erfc}\left(\sqrt{\frac{RP_1 ER}{q\Delta f}}\frac{(1 - 1/ER)}{2}\right) \tag{12}$$

Supplementary Fig. 6(b) shows the achievable BER as a function of ER for a shot-noise limited detector with bandwidth Δf = 100 MHz, assuming a responsivity R = 0.5 A/W, for different '1' bit received optical powers P_1. Clearly, ER in the order of 0.5 dB are enough to achieve error-free communication for received powers above 1 μW.

[3] The expression for the BER in Supplementary Eq. (12) assumes that the optimal decision threshold I_{th} corresponds to the mid-point between the received current for bit '1' ($I_1 = RP_1$) and the received current for bit '0' ($I_0 = RP_0$): $I_{\text{th}} = 0.5(I_0 + I_1)$. This is strictly correct only in the case where the noise power is the same for bit '0' and '1', which is in general not true for a shot noise limited system. Nevertheless, for low enough ER such as the ones considered here this is a good approximation.

It is worth noting that the SNR achieved in the experimental waveforms shown in Fig. 4 in the main text are lower than the achievable SNR based on the calculations outlined above. This is due to the high optical losses associated to the coupling of light in and out of the chip. Because we use a non-optimized, broadband design, each grating coupler adds ~ 10 dB insertion loss to the optical path. Since the signal power decreases but the total noise (dominated by the receiver electrical noise) is not affected, this additional insertion loss translates into a decrease in SNR: a 10 dB loss output coupler results in a 10 dB decrease in our experimentally achieved SNR compared to what we could achieve if we were to use ideal couplers. For instance, based on the experimentally achieved SNR of the waveform in Fig. 4(e) (with an ER = 0.6 dB and P_1 =3.75 μW) we expect a BER ≈ $3\cdot10^{-9}$. If instead we use Supplementary Eq. (11) for this ER and received optical power, a BER < 10^{-13} could be realized.

This is a limitation of the chip design and the testing setup – not an intrinsic limitation of the device or the fabrication technology. We have demonstrated grating couplers with over 90% coupling efficiency (0.5 dB loss) in the same technology platform we use to fabricate our photovoltaic modulator devices (GlobalFoundries 45RF SOI) [23].

Supplementary Discussion 7. PV modulation in a Si-only device.

The experimentally measured device presented in the main text includes a ring of SiGe to enhance optical absorption and increase device responsivity and photocurrent generation, which as we have discussed results in a faster device.

Nevertheless, as discussed in detail in Supplementary Discussion 1 and experimentally characterized in Supplementary Discussion 2, Si-only optical modulators have an intrinsic photovoltaic response dominated by surface state absorption, which results in responsivities of approximately 1 mA/W. Thus, photovoltaic modulation can be leveraged in Si-only devices.

We experimentally demonstrated PV modulation in a Si-only device designed for operation at wavelengths around 1550 nm, fabricated in the same 45 nm CMOS SOI process (see [16] for the device design and characterization). As presented in Supplementary Discussion 2, this device has a measured responsivity R = 0.85 mA/W and develops open circuit voltages above 0.5 V for on-chip input optical powers larger than 20 μW (Supplementary Fig. 1).

Supplementary Fig. 7 shows the DC transmission spectra of the Si-only modulator operated in PV mode in the open circuit (V_{in} = 0, where the transistor is turned off) and short circuit conditions (V_{in} =0.7 V, where the transistor is turned on). As expected, the resonance wavelength blue shifts when the transistor is turned on due to a decrease in V_{mod} that is accompanied by a decrease in the free-carrier density. Two different on-chip optical powers of 280 μW (Supplementary Fig. 7(a)) and 900 μW (Supplementary Fig. 7(b)) are shown. Notice how the resonance wavelength shift is larger for the larger input optical power of 900 μW. This is because a larger optical power results in a larger generated photocurrent I_{pc} and thus a larger open circuit voltage V_{oc}.

Supplementary Discussion 8. The effect of r_{pc}.

In the analysis presented in this work we have assumed that the equivalent resistance associated to the change in photocurrent with the voltage at the modulator terminals $r_{\mathrm{pc}} = \left(dI_{\mathrm{pc}}/dV_{\mathrm{mod}}\right)^{-1}$ is much larger than the diode dynamic resistance r_d and the transistor output resistance r_0, so that we can say $r_d||r_0||r_{\mathrm{pc}} \approx r_d||r_0$. It is pertinent to study the validity of this assumption and study how r_{pc} could affect the operation of a PV modulator.

In any optical modulator used in PV modulation, a change in the voltage at the modulator terminals V_{mod} will be accompanied by a change in the photogenerated current I_{pc}. Take, for example, a resonant modulator as the one we used in our experiments. A change in V_{mod} will shift the resonance wavelength of the device, modifying the power absorbed in the ring and resulting in a change in I_{pc}. Consequently, $r_{\mathrm{pc}} = \left(dI_{\mathrm{pc}}/dV_{\mathrm{mod}}\right)^{-1}$ has a finite value and can affect the performance of a PV modulator.

It is easy to derive an expression for the value of r_{pc}, recognizing that the generated photocurrent is given by $I_{\mathrm{pc}} = RP_{\mathrm{abs}}$, where R is the device responsivity and P_{abs} is the optical power absorbed in the modulator. We can then write:

$$\frac{dI_{\mathrm{pc}}}{dV_{\mathrm{mod}}} = R\frac{dP_{\mathrm{abs}}}{dV_{\mathrm{mod}}} = RP_{\mathrm{in}}\frac{d(1-T)}{dV_{\mathrm{mod}}} = -RP_{\mathrm{in}}\frac{dT}{d\lambda}\frac{d\lambda}{dV_{\mathrm{mod}}} \quad (13)$$

Where $T = P_{\mathrm{out}}/P_{in}$ is the transmission through the ring, and we have used $P_{\mathrm{abs}} = P_{\mathrm{in}}(1-T)$. We can rewrite the term $d\lambda/dV_{\mathrm{mod}} = -d\lambda_0/dV_{\mathrm{mod}}$, which is the intrinsic modulation efficiency of our modulator.

We end up with the following expression for r_{pc}:

$$r_{\mathrm{pc}} = \frac{1}{\frac{dI_{\mathrm{pc}}}{dV_{\mathrm{mod}}}} = \left(RP_{\mathrm{in}}\frac{dT}{d\lambda}\frac{d\lambda_0}{dV_{\mathrm{mod}}}\right)^{-1} \quad (14)$$

Supplementary Fig. 8 shows the calculated r_{pc} using Supplementary Eq. (14) for our resonant modulator under different input optical powers, as a function of laser wavelength. We used a responsivity R = 0.034 A/W, assumed a modulation efficiency $d\lambda_0/dV_{\mathrm{mod}}$ = - 20 pm/V and a Lorentzian resonance with Q_{coup} = 20,000, Q_{int} = 9,000 and λ_0 = 1270 nm. Several important observations should be made:

1. For a given input optical power, the minimum achievable values of r_{pc} are about 10x larger than typical values for $r_d||r_0$ (see for example Supplementary Fig. 2(d)). This confirms that our assumption $r_d||r_0||r_{\text{pc}} \approx r_d||r_0$ is correct for our device.
2. Negative r_{pc} values can be obtained when the laser wavelength is at the red side of the resonance ($\lambda_l > \lambda_0$). This is because in a Lorentzian resonance, $dT/d\lambda > 0$ for $\lambda_l > \lambda_0$.

While in our experimental demonstration $r_{\text{pc}} \gg r_0 \, ||r_d$, a scenario where r_{pc} becomes comparable to r_0 (or even $r_{\text{pc}} < r_0 \, ||r_d$) is possible by, for example, using a modulator with a larger modulation efficiency $d\lambda_0/dV_{\text{mod}}$ or a sharper resonance (and therefore larger $dT/d\lambda$). In this case, we would need to account for r_{pc} when computing the small signal gain and bandwidth, resulting in the following expressions:

$$A_v = g_m\left(r_0||r_d||r_{\text{pc}}\right) \tag{15}$$

$$f_{3dB} = \frac{1}{2\pi C_{\text{diode}}\left(r_0||r_d||r_{\text{pc}}\right)} \tag{16}$$

And of course:

$$r_0||r_d||r_{\text{pc}} = \frac{r_{\text{pc}}\,(r_0||r_d)}{r_{\text{pc}} \, + \, (r_0||r_d)} \tag{17}$$

Clearly, if we operate a device where $r_{\text{pc}} \ll r_0 \, ||r_d$, then $r_d||r_0||r_{\text{pc}} \approx r_{\text{pc}}$. This would result in a reduced small signal gain A_V than that predicted by our simulations, but an increased frequency response f_{3dB}.

An interesting situation arises when we operate at a bias point where $r_{\text{pc}} \approx -r_0 \, ||r_d$. In this case, $r_0||r_d||r_{\text{pc}} \rightarrow \infty$, which makes $A_V \rightarrow \infty$ and $f_{3d} \;\; \rightarrow 0$. The possibility of synthesizing negative resistances is interesting for other applications beyond modulation, such as the realization of on chip oscillators.

We can thus conclude that r_{pc} can have a significant effect in the PV modulator behavior if the photogenerated current depends strongly on the voltage applied at the modulator terminals. This was not the case for the device we experimentally demonstrated here, but it could be important in other devices with increased modulation efficiency or higher Q factor.

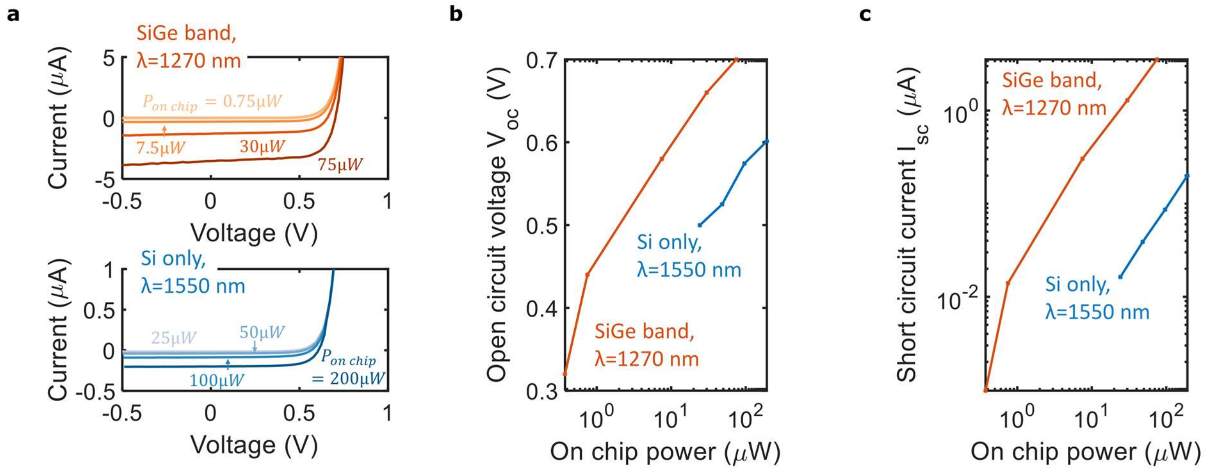

Supplementary Fig. 1. Photocurrent generation in silicon optical modulators. (**a**) IV curve, (**b**) open circuit voltage and (**c**) short circuit current under different on-chip input optical powers for two different resonant optical modulators. Blue shaded curves correspond to a Si-only modulator operating at 1550 nm [16] and orange curves to the modulator with a silicon-germanium (SiGe) band operating at 1270 nm, which we used for the experimental demonstration of photovoltaic modulation reported here.

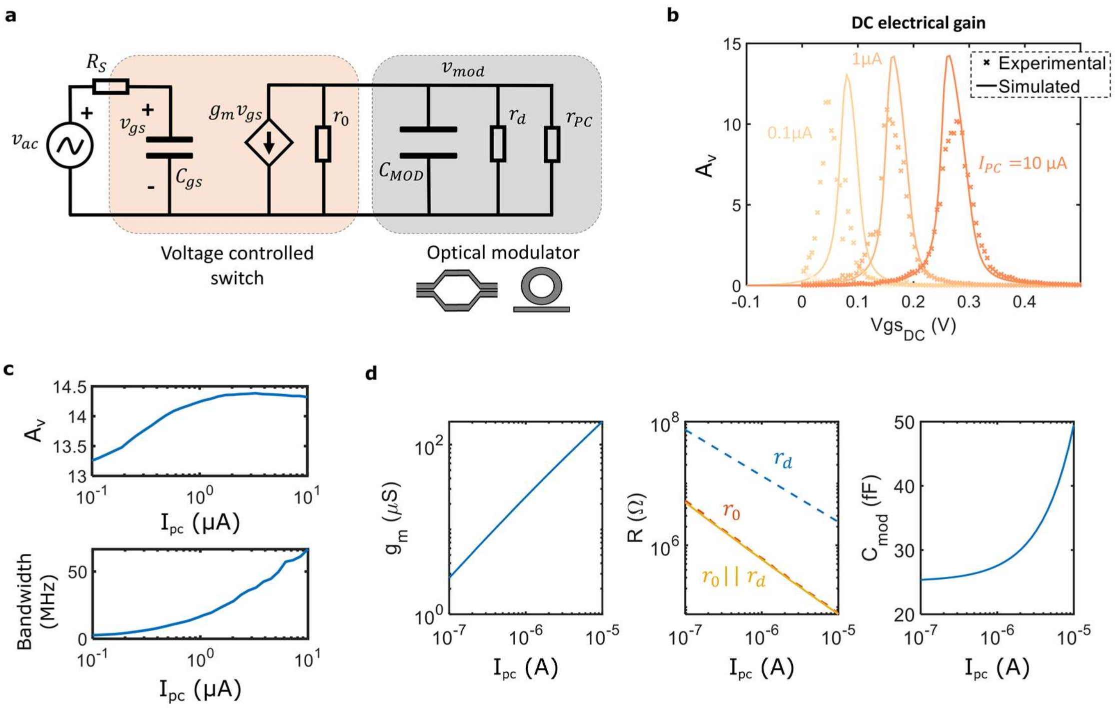

Supplementary Fig. 2. Photovoltaic modulator characteristics at the maximum gain point. (**a**) Small signal equivalent circuit model for the photovoltaic (PV) modulator. (**b**) Measured (crosses) and simulated (lines) voltage gain as a function of gate bias for different photocurrents I_{pc}. (**c**) Gain (top) and bandwidth (bottom) at the maximum gain point as a function of photocurrent. (**d**) Evolution of the transistor transconductance g_m (left), system resistance R (middle; r_0 is the output resistance of the transistor and r_d the dynamic resistance of the modulator) and modulator capacitance C_{mod} (right) with photocurrent at the maximum gain point.

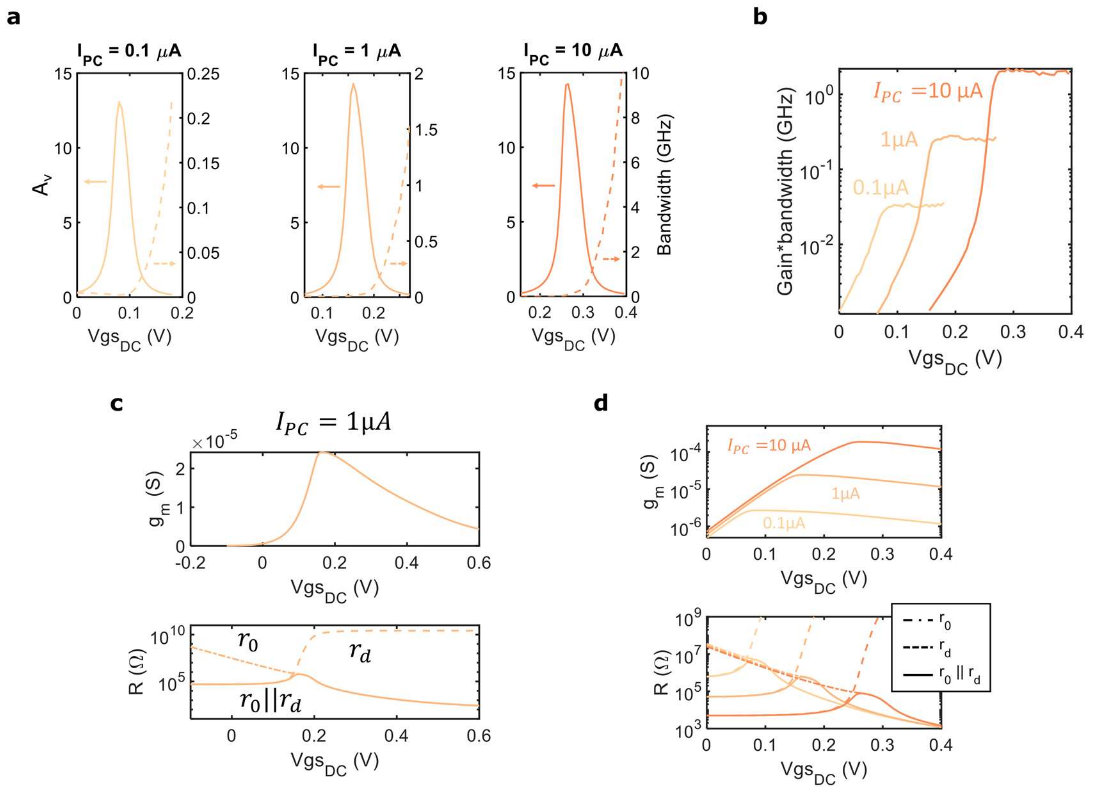

Supplementary Fig. 3. Gain-bandwidth tradeoff in a photovoltaic modulator. (**a**) Gain (solid line, left axis) and 3 dB bandwidth (dashed line, right axis) as a function of gate bias ($V_{\mathrm{gs,DC}}$) for 0.1 μA (left), 1 μA (middle) and 10 μA (right) generated photocurrent (I_{pc}). (**b**) Gain-bandwidth product as a function of bias voltage for the same photocurrents as in (**a**). (**c**) Evolution of the transistor transconductance g_m (top) and the system resistances (bottom) as a function of gate bias for a 1 μA photocurrent. r_0 is the output resistance of the transistor and r_d the dynamic resistance of the modulator. (**d**) Evolution of g_m (top) and the system resistances (bottom) as a function of gate bias for the same photocurrents as (**a**) and (**b**).

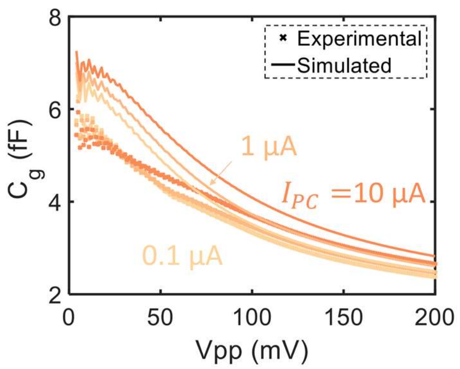

Supplementary Fig. 4. Equivalent gate capacitance C_g in photovoltaic modulators. Solid lines correspond to C_g inferred from simulation results, while crosses correspond to C_g inferred using experimentally measured electrical gain. Different colors correspond to different photocurrents (I_{pc}).

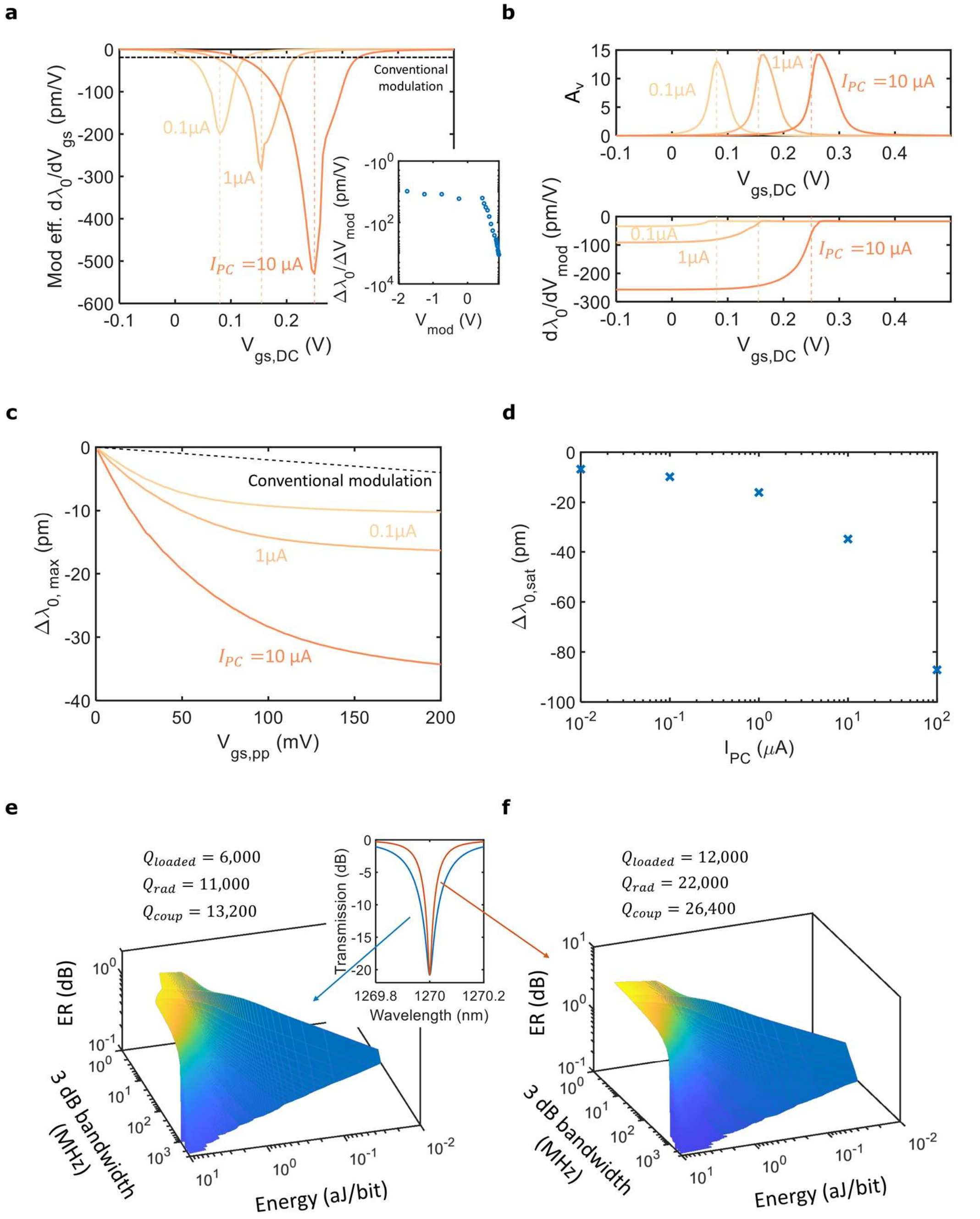

Supplementary Fig. 5. Modulation performance in a photovoltaic modulator. Caption in next page.

Supplementary Fig. 5. Modulation performance in a photovoltaic modulator. (**a**) Modulation efficiency from the photovoltaic (PV) modulator input terminals ($d\lambda_0/dV_{gs}$) as a function of gate bias voltage ($V_{\mathrm{gs,DC}}$). The dashed black line shows the modulation efficiency in conventional modulation. The inset shows the intrinsic modulation efficiency of the modulator $d\lambda_0/dV_{\mathrm{mod}}$ used for the calculations, which is based on experimental results. (**b**) Top: Electrical gain A_v as a function of gate bias voltage. Bottom: Intrinsic modulation efficiency $d\lambda_0/dV_{\mathrm{mod}}$ as a function of gate bias voltage. For (**a**) and (**b**), the dashed lines denote the point of maximum modulation efficiency. (**c**) Maximum achievable resonance shift for the PV modulator as a function of input peak to peak voltage. The dashed black line shows the resonance shift of conventional modulation. (**d**) Saturation wavelength shift as a function of photocurrent (I_{pc}). Regardless of input peak to peak voltage, this is the maximum wavelength shift achievable in a PV modulator. (**e, f**) The ER as a function of electrical energy dissipation and 3 dB bandwidth is shown for a resonator with Q_{loaded} = 6,000 (**e**) and for Q_{loaded} = 12,000 (**f**). The resonance shape $T(\lambda)$ is shown in the inset in the middle and the photocurrent is I_{pc} = 1 μA.

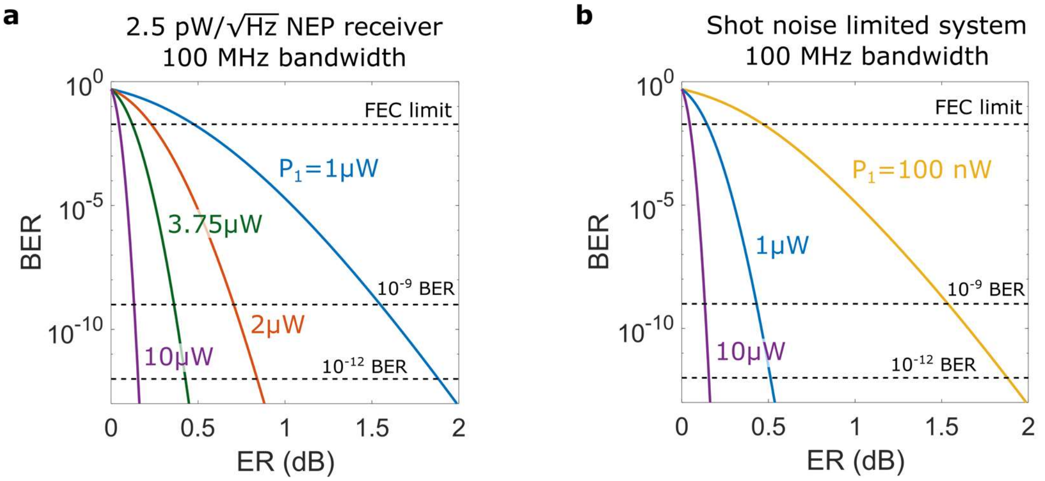

Supplementary Fig. 6. Bit error rate (BER) for low speed, low extinction ratio (ER) communication links. (**a**) BER as a function of ER for a 100 MHz communication link where the signal-to-noise ratio is limited by the receiver noise. A 2.5 pW/$\sqrt{Hz}$ noise equivalent power (NEP), representative of commercially available receivers, is considered. (**b**) BER as a function of ER for a 100 MHz, shot-noise limited communication link. For both (**a**) and (**b**), different colors show different received optical powers for the '1' bit, P_1. The necessary BER for 3rd generation forward error correction (FEC), as well as the 10^{-9} and 10^{-12} BER level (considered error free in most telecommunications and data communications standards, respectively), are depicted in black.

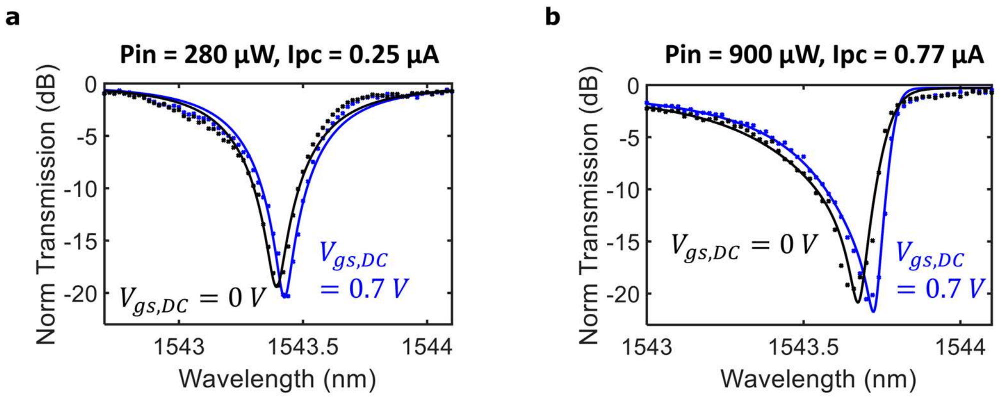

Supplementary Fig. 7. Resonance wavelength shift due to the photovoltaic effect in a Si-only modulator. DC transmission spectra of a Si-only modulator [16] operated in the photovoltaic mode. (**a**) 280 μW on-chip input optical power, corresponding to a 0.25 μA generated photocurrent. (**b**) 900 μW on-chip input optical power, corresponding to a 0.77 μA generated photocurrent. The black curves correspond to the device biased in the open circuit condition (transistor turned off) and the blue curves to the short circuit condition (transistor turned on). The shift in resonance wavelength between the two bias points is clear.

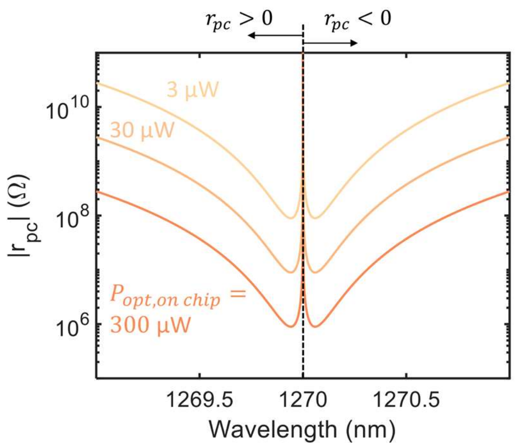

Supplementary Fig. 8. r_{pc} in resonant modulators. r_{pc} as a function of laser wavelength for the device used in the experimental demonstration of the PV modulator. Different on chip input optical powers are shown.

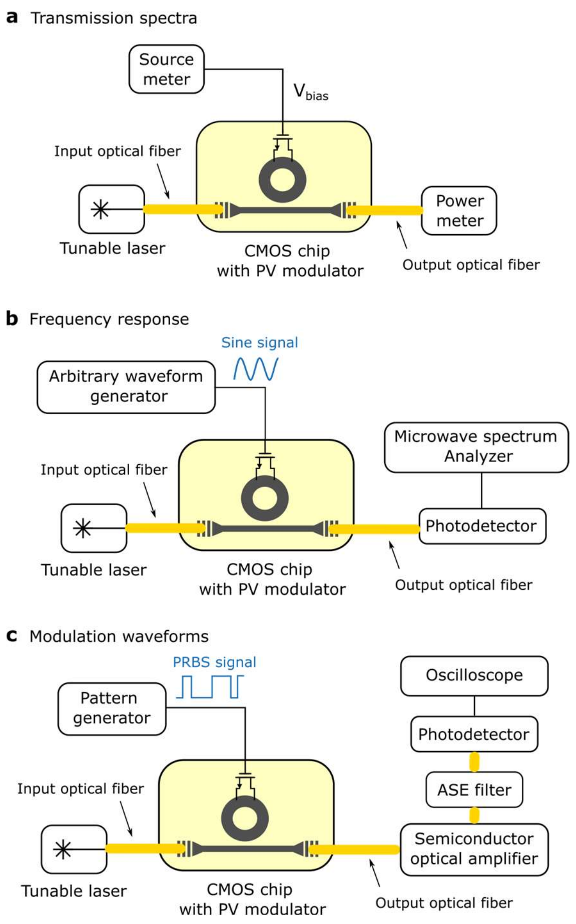

Supplementary Fig. 9. Experimental setups. (**a**) Setup used for recording transmission spectra. The transmission through the device is recorded as the laser wavelength is swept. (**b**) Setup used for measuring the bandwidth of the device. By sweeping the frequency of the driving sinusoid and recording the power of the optical signal at that same frequency through a microwave spectrum analyzer, the frequency response of the device is obtained. (**c**) Setup used for recording modulation waveforms. Optical amplification is required to generate a large enough signal to be observed in the oscilloscope. ASE = Amplified Spontaneous Emission.

Supplementary Table 1. Low power Si optical modulator demonstrations.

Fig. 2(a) in the main text shows the lowest energy dissipation optical modulator of its kind. Supplementary Table 1 shows extended information on the silicon modulators shown there, as well as other silicon optical modulators reported in the literature.

Ref., Year	Structure	Electrical energy	Bandwidth	Eye diagram demonstrations				$P_{opt, on chip}$	Optical energy
				Speed	V_{pp}, V_{bias}	ER	IL*		
[24] 2014	MRR, reverse biased vertical p-n junction	0.9 fJ/bit	21 GHz	25 Gbps	0.5 V_{pp} V_{bias} NR	6.1dB	1dB	-1.75 dBm	5.5 fJ/bit
[25] 2020	MRR, forward biased interleaved p-n junction	2 fJ/bit	0.9 GHz	2 Gbps	0.2V_{pp} 0.7 V_{bias}	2.6dB	5.7dB	0 dBm	360 fJ/bit
[26] 2019	MRR, reverse biased p-n junction	3 fJ/bit †	NR	32 Gbps	0.75 V_{pp} V_{bias} NR	3.5dB	3.1dB	NR	NR
[27] 2015	MRR, reverse biased p-n junction	10 fJ/bit	38 GHz	40 Gbps	1.5 V_{pp} -0.25 V_{bias}	4dB	3dB	NR	NR
[28] 2013	MZM, zero bias lateral p-n junction	32.4 fJ/bit	12.5 GHz	40 Gbps	0.36 V_{pp} 0 V_{bias}	3.2dB	3dB	NR	NR
[29] 2009	MRR, reverse biased lateral p-n junction	50 fJ/bit	11 GHz	10 Gbps	2 V_{pp} -1 V_{bias}	6.5dB	2dB	NR	NR
[16] 2016	MRR, reverse biased lateral p-n junction	70 fJ/bit	14 GHz	25 Gbps	3.6 V_{pp} -2 V_{bias}	6.2dB	0.5dB	-3 dBm	2 fJ/bit
[30] 2009	MRR, forward biased lateral p-i-n junction	86 fJ/bit	NR	3 Gbps	0.5 V_{pp} 1.4 V_{bias}	NR	NR	-2.2 dBm	74 fJ/bit ‡
[31] 2013	MZM, forward biased lateral p-i-n junction	900 fJ/bit	200 MHz	50 Gbps	4.35 V_{pp} 0.5 V_{bias}	4.3dB	7.2dB	NR	NR
[32] 2014	MZM, vertical MOS capacitor	2 pJ/bit	NR	40 Gbps	1 V_{pp} 1.7 V_{bias}	8dB	NR	NR	NR
[33] 2018	MZM, lateral MOS capacitor	28 pJ/bit	NR	25 Gbps	6 V_{pp} 3 V_{bias}	3.6dB	2.5dB	NR	NR

* Does not account for the losses associated with coupling light into the chip

† Assuming C_{in} = 20 fF

‡ Assuming 2 dB IL

Supplementary Table 1. Low power silicon optical modulators reported in the literature, ordered from lowest to highest electrical energy dissipation. NR = Not reported. MRR = Micro-ring resonator. MZM = Mach-Zehnder Modulator. ER = Extinction Ratio. IL = Insertion Loss.

Supplementary Table 2. Low power non-Si optical modulator demonstrations.

Supplementary Table 2 shows extended information on the non-silicon modulators shown in Fig. 2(a) in the main text, as well as other non-silicon optical modulators reported in the literature.

Ref., Year	Material	Electrical energy	Bandwidth	Eye diagram demonstrations				$P_{opt, on chip}$	Optical energy
				Speed	V_{pp}, V_{bias}	ER	IL*		
[34] 2018	$LiNbO_3$	37 aJ/bit	45 GHz	70 Gbps	60 mV_{pp} V_{bias} NR	3.4e-3 BER	0.4dB	8 dBm	200 aJ/bit
[35] 2019	InGaAsP photonic crystal	42 aJ/bit	45 GHz	40 Gbps	0.5 V_{pp} V_{bias} NR	2.4dB	2.3dB	-10 dBm	1 fJ/bit
[36] 2019	Plasmonic-Organic hybrid	70 aJ/bit	NR	50 Gbps	145 mV_{pp} V_{bias} NR	2e-3 BER	11.2 dB	8 dBm	116 fJ/bit
[37] 2015	Electro-optic polymer	12 fJ/bit	NR	12.5 Gbps	0.3 V_{pp} V_{bias} NR	NR	6dB	-0.25 dBm	56 fJ/bit
[38] 2018	Plasmonic	12 fJ/bit	>100 GHz	72 Gbps	3.3 V_{pp} V_{bias} NR	1e-3 BER	2.5dB	-3 dBm	3 fJ/bit
[39] 2012	InP	43 fJ/bit	NR	10 Gbps	1.62 V_{pp} -1.6 V_{bias}	2.2 dB	NR	-10 dBm	3.7 fJ/bit ‡
[40] 2017	Plasmonic	110 fJ/bit	>70 GHz	72 Gbps	5.6 V_{pp} V_{bias} NR	5.5e-6 BER	6dB	15 dBm	33 pJ/bit
[41] 2012	SiGe	240 fJ/bit	40 GHz	28 Gbps	2.8 V_{pp} 1.4 V_{bias}	5.9dB	4dB	NR	NR
[42] 2016	Graphene	350 fJ/bit	5.9 GHz	10 Gbps	2.5 V_{pp} 1.75 V_{bias}	2.3dB	3.8dB	NR	NR
[43] 2017	InP	400 fJ/bit	>67 GHz	100 Gbps	2.3 V_{pp} -5 V_{bias}	10dB	2dB	9.5 dBm	33 fJ/bit

* Does not account for the losses associated with coupling light into the chip

‡ Assuming 2 dB IL

Supplementary Table 2. Low power non-Si optical modulators reported in the literature, ordered from lowest to highest electrical energy dissipation. NR = Not reported. ER = Extinction Ratio. IL = Insertion Loss.

Supplementary Table 3. SPICE diode parameters.

Supplementary Table 3 shows the diode model parameters used in the simulation of the photovoltaic (PV) modulator. These correspond to experimental values measured on the SiGe CMOS modulator used for the experimental demonstration of the PV modulator concept, but similar values are obtained for other devices such as the Si only modulator shown in Supplementary Fig. 1. Supplementary Table 3 also contains a brief note on how the model parameters were measured.

Parameter	Description	Value	Extracted from
I_s	Reverse saturation current	1.81×10^{-12} A	Fit to IV curve
R_s	Series resistance	0.5 Ω	Fit to IV curve
n	Ideality factor	1.92	Fit to IV curve
C_{j0}	Zero bias junction capacitance	20 fF	Parasitic extraction from layout
V_j	Junction potential	0.95 V	Estimated from doping concentrations of p and n regions
m	Grading coefficient	0.4	---
TT	Transit time	45 ns	Fit to falling edge of modulation waveform in forward bias conditions
R *	Responsivity	0.034 A/W	Photocurrent vs input power (Supplementary Fig. 1(c))

* This is not a SPICE diode model parameter

Supplementary Table 3. Spice diode model parameters used to describe the electrical behavior of the modulator.

Supplementary Table 4. Estimated Surface State Absorption coefficient α_{SSA} in silicon photonic waveguides from published works. MRR = Micro-ring resonator.

Ref.	Structure	Responsivity	η	α_{SSA}
[44]	Straight Si waveguide with air cladding, 500 x 100 nm cross section	36 mA/W (low input power)<br>1.5 mA/W (high input power)	2.8 %<br>0.12 %	19 m^{-1}<br>0.8 m^{-1}
[10]	Straight SOI waveguide, 450 x 220 nm cross section	---	---	1.9 m^{-1}
[45]	MRR, lateral p-n junction in SOI waveguide, 500 x 200 nm cross section	1 mA/W	0.08 %	2.22 m^{-1}

Supplementary Table 5. Surface State Absorption (SSA) photocurrent generation in state-of-the-art silicon modulators. The last column shows the percentage of the total electrical power consumption that is due to photocurrent. More information on these modulators is shown in Supplementary Table 1. MRR = Micro-ring resonator. MZM = Mach-Zehnder modulator.

Ref., Year	Structure	Responsivity	$P_{in, opt}$	I_{pc}	V_{bias}	E_{DC} @ f	$E_{DC}/E_{el,TOT}$
[24] 2014	MRR, reverse biased vertical p-n junction	1 mA/W	-1.75 dBm	0.66 µA	-1 V *	25 aJ/bit @ 25 Gbps	2.25 %
[46] 2016	MZM, reverse biased lateral p-n junction	3.75 mA/W	12 dBm	60 µA	-0.8 V	2 fJ/bit @ 25 Gbps	6.25 %
[30] 2009	MRR, forward biased lateral p-i-n junction	1 mA/W	-2.2 dBm	0.6 µA	1.4 V	- 280 aJ/bit @ 3 Gbps	0.32 %

* Estimated

SUPPLEMENTARY REFERENCES

[1] Casalino, M. & Coppola, G. & Iodice, M. & Rendina, I. & Sirleto, L. Near-infrared sub-bandgap all-silicon photodetectors: state of the art and perspectives. *Sensors* **10**, 10571-10600 (2010).

[2] Morichetti, F. et al. Non-invasive on-chip light observation by contactless waveguide conductivity monitoring. *IEEE Journal of Selected Topics in Quantum Electronics* **20**, 292-301 (2014).

[3] Yu, H. et al. Using carrier-depletion silicon modulators for optical power monitoring. *Optics Letters* **37**, 4681-4683 (2012).

[4] Jayatilleka, H. et al. Wavelength tuning and stabilization of microring-based filters using silicon in-resonator photoconductive heaters. *Optics Express* **23**, 25084-25097 (2015).

[5] Li, X. et al. 40 Gb/s All-Silicon Photodetector Based on Microring Resonators. *IEEE Photonics Technology Letters* **27**, 729-732 (2015).

[6] Geis, M. W et al. CMOS-compatible all-Si high-speed waveguide photodiodes with high responsivity in near-infrared communication band. *IEEE Photonics Technology Letters* **19**, 152-154 (2007).

[7] Zhang, P. et al. Electronic transport in nanometre-scale silicon-on-insulator membranes. *Nature* **439**, 703-706 (2006).

[8] Grillanda, S. & Morichetti, F. Light-induced metal-like surface of silicon photonic waveguides. *Nature Communications* **6**, 8182 (2015).

[9] Li, Y. & Poon, A. W. Characterization of surface-state absorption in foundry-fabricated silicon ridge waveguides at 1550 nm using photocurrents. *2016 Conference on Lasers and Electro-Optics (CLEO)* (2016).

[10] Gil-Molina, A. et al. Optical free-carrier generation in silicon nano-waveguides at 1550 nm. *Applied Physics Letters* **112**, 251104 (2018).

[11] Borselli, M. & Johnson, T. J. & Painter, O. Measuring the role of surface chemistry in silicon microphotonics. *Applied Physics Letters* **88**, 131114 (2006).

[12] Chiarotti, G. & Nannarone, S. & Pastore, R. & Chiaradia, P. Optical absorption of surface states in ultrahigh vacuum cleaved (111) surfaces of Ge and Si. *Physical Review B* **4**, 3398-3402 (1971).

[13] Tsakalakos, L. et al. Strong broadband optical absorption in silicon nanowire films. *Journal of Nanophotonics* **1**, 1-10 (2007).

[14] Alloatti, L & Ram, R. J. Resonance-enhanced waveguide-coupled silicon-germanium detector. *Applied Physics Letters* **108**, 071105 (2016).

[15] Braunstein, R. & Moore A. R. & Herman, F. Intrinsic Optical Absorption in Germanium-Silicon Alloys. *Phyical Review* **109**, 695-710 (1958).

[16] de Cea, M. et al. A Thin Silicon Photonic Platform for Telecommunication Wavelengths. *2017 European Conference on Optical Communication (ECOC)* (2017).

[17] Razavi, B. *Design of Analog CMOS Integrated Circuits* (McGraw Hill, 2017).

[18] Soref, R. & Bennett, B. Electrooptical effects in silicon. *IEEE Journal of Quantum Electronics* **23**, 123-129 (1987).

[19] Newport model 1811-FS optical receiver [Online]. Available: https://www.newport.com/p/1811-FS.

[20] Thorlabs FPD510-FS-NIR amplified photodetector [Online]. Available: https://www.thorlabs.us/thorproduct.cfm?partnumber=FPD510-FS-NIR.

[21] Texas Instruments OPA857 transimpedance amplifier [Online]. Available: https://www.ti.com/product/OPA857.

[22] Analog Devices LTC6561 transimpedance amplifier [Online]. Available: https://www.analog.com/en/products/ltc6561.html.

[23] Notaros, J. et al. Ultra-efficient CMOS fiber-to-chip grating couplers. *2016 Optical Fiber Communication Conference (OFC)* (2016).

[24] Timurdogan, E. et al. An ultralow power athermal silicon modulator. *Nature Communications* **5**, 4008 (2014).

[25] de Cea, M. et al. Photonic readout of superconducting nanowire single photon counting detectors. *Scientific Reports* **10**, 9470 (2020).

[26] Timurdogan, E. et al. APSUNY Process Design Kit (PDKv3.0): O, C and L Band Silicon Photonics Component Libraries on 300mm Wafers. *Optical Fiber Communication Conference (OFC) 2019 (*2019).

[27] Pantouvaki, M. et al. 56Gb/s ring modulator on a 300mm silicon photonics platform. *2015 European Conference on Optical Communication (ECOC) (*2015).

[28] Ding, J. & Ji, R. & Zhang, L. & Yang, L. Electro-Optical response analysis of a 40 Gb/s silicon Mach-Zehnder optical modulator. *Journal of Lightwave Technology* **31**, 2434-2440 (2013).

[29] Dong, P. et al. Low Vpp, ultralow-energy, compact, high-speed silicon electro-optic modulator. *Optics Express* **17**, 22484-22490 (2009).

[30] Chen, L. & Preston, K. & Manipatruni, S. & Lipson, M. Integrated GHz silicon photonic interconnect with micrometer-scale modulators and detectors. *Optics Express* **17**, 15248-15256 (2009).

[31] Akiyama, S. et al. Compact PIN-Diode-Based Silicon Modulator Using Side-Wall-Grating Waveguide. *IEEE Journal of Selected Topics in Quantum Electronics* **19**, 74-84 (2013).

[32] Webster, M. et al. An efficient MOS-capacitor based silicon modulator and CMOS drivers for optical transmitters. *11th International Conference on Group IV Photonics (GFP)* (2014).

[33] Debnath, K. et al. All-silicon carrier accumulation modulator based on a lateral metal-oxide-semiconductor capacitor. *Photonics Research* **6**, 373-379 (2018).

[34] Wang, C. et al. Integrated lithium niobate electro-optic modulators operating at CMOS-compatible voltages. *Nature* **562**, 101-104 (2018).

[35] Nozaki, K. et al. Femtofarad optoelectronic integration demonstrating energy-saving signal conversion and nonlinear functions. *Nature Photonics* **13**, 454-459 (2019).

[36] Heni, W. et al. Plasmonic IQ modulators with attojoule per bit electrical energy consumption. *Nature Communications* **10**, 1694 (2019).

[37] Koeber, S. et al. Femtojoule electro-optic modulation using a silicon-organic hybrid device. *Light: Science & Applications* **4**, e255 (2015).

[38] Haffner, C. et al. Low-loss plasmon-assisted electro-optic modulator. *Nature* **556**, 483-486 (2018).

[39] Hofrichter, J. et al. A low-power high-speed InP microdisk modulator heterogeneously integrated on a SOI waveguide. *Optics Express* **20**, 9363-9370 (2012).

[40] Ayata, M. et al. High-speed plasmonic modulator in a single metal layer. *Science* **358**, 630-632 (2017).

[41] Feng, D. et al. High speed GeSi electro-absorption modulator at 1550 nm wavelength on SOI waveguide. *Optics Express* **20**, 22224-22232 (2012).

[42] Hu, Y. et al. Broadband 10 Gb/s operation of graphene electro-absorption modulator on silicon. *Laser & Photonics Reviews* **10**, 307-316 (2016).

[43] Ogiso, Y. et al. Over 67 GHz Bandwidth and 1.5 V Vpi InP-Based Optical IQ Modulator With n-i-p-n Heterostructure. *Journal of Lightwave Technology* **35**, 1450-1455 (2017).

[44] Baehr-Jones, T. & Hochberg, N. & Scherer, A. Photodetection in silicon beyond the band edge with surface states. *Optics Express* **16**, 1659-1668 (2008).

[45] Li, Y. & Feng, S. & Zhang, Y & Poon, A. W. Sub-bandgap linear-absorption-based photodetectors in avalanche mode in PN-diode-integrated silicon microring resonators. *Optics Letters* **38**, 5200-5203 (2013).

[46] Xiong, C. et al. Monolithic 56 Gb/s silicon photonic pulse-amplitude modulation transmitter. *Optica* **3**, 1060-1065 (2016).
